# Knowledge, Attitudes and Practices towards Sexual and Reproductive Health and Rights of Girls among Colombian Healthcare Professionals

**DOI:** 10.3390/ijerph191912295

**Published:** 2022-09-28

**Authors:** Aura Y. Rodríguez-Burbano, Diana M. Galván-Canchila, Rocío de Diego-Cordero

**Affiliations:** 1 Facultad de Ciencias Sociales, Universidad de Santander, Bucaramanga 680003, Colombia; 2 Facultad de Ciencias Médicas y de la Salud, Universidad de Santander, Bucaramanga 680003, Colombia; 3 Research Group CTS 969 Innovation in Healthcare and Social Determinants of Health, University of Seville, 41009 Seville, Spain

**Keywords:** healthcare professionals, sexual and reproductive rights, gender-based violence, violence against women, vulnerable populations

## Abstract

This research aims to determine knowledge and attitudes towards sexual and reproductive health and rights of adolescent girls among healthcare professionals working at Café Madrid and Colorados health centers, which are highly vulnerable neighborhoods in Bucaramanga, Santander. To this end, in-depth interviews were conducted with a total of eight healthcare professionals from the above health centers using a script based on WHO recommendations on adolescent sexual and reproductive health and rights and recommendations by the DAPRE-Presidential Council for Women’s Equity—CedaVida Foundation. Healthcare professionals were found to have proper technical and legal knowledge, especially regarding comprehensive care packages for survivors, as well as a gender perspective in their professional practice aimed at achieving equity. Knowledge and experience with sexual and reproductive health and rights provide insight into women’s health from unrestrained choice of contraceptive methods to procedures such as abortion within the current legal framework. Their professional work is also affected by potential barriers that may limit their actions when putting their knowledge into practice.

## 1. Introduction

According to the World Health Organization. (WHO), gender-based violence against girls and adolescents is a persistent problem [1]. Twenty-nine percent of adolescent girls who got married before age 19 have experienced intimate partner violence. Regarding children, 29% of them have been survivors of sexual violence worldwide, of which 18% are girls [2,3]. In Colombia, according to the Legal Medicine Institute, 18,043 cases of sexual crimes were reported in 2020, of which 15,359 cases involved children and adolescents aged 0 to 17, accounting for 85.1% of all sexual aggressions [4].

Gender-based violence by gender inequality is present in diverse settings such as the home, public spaces and even at school. The WHO indicates that gender-based violence against adolescent girls has both health and social consequences. Intimate partner violence may lead to unwanted pregnancy, induced abortion and sometimes HIV and sexually transmitted diseases. In terms of mental health, sexual abuse may lead to depression and suicidal ideation. Moreover, sexual abuse may lead to risky behaviors such as alcohol abuse and unprotected sex during adolescence and adulthood. In addition, girls and women who have experienced gender-based violence do not report it or seek medical care as they are afraid of being stigmatized and ashamed. In this light, healthcare professionals, who are probably the first group that survivors of sexual violence would seek for, are not adequately prepared to deal with this type of violence, in addition to an insufficient coordination between health programs and services, which impedes addressing violence experienced by girls and women in an effective manner [1].

According to the Guidelines for health professionals in responding to women facing violence, the lack of coordination among actors involved, combined with excessive specialized functions, may sometimes hinder care and referral of girls and women survivors of violence. In addition, this guideline highly recommends the participation of additional actors, such as the police, judicial and social services, and organizations working with communities so that multisectoral training can help ensure that all participants share the same understanding of the necessary steps to address healthcare needs of women survivors, the dos and don’ts of medical–legal assistance provided by healthcare professionals and strengthening coordination, referrals and community support for women survivors of violence [5].

Considering the above issues, the WHO has made several recommendations to address sexual violence against girls and adolescents, among which one related to healthcare personnel led us to the development of this research. The WHO has been advocating for the training of healthcare personnel to deal with this type of violence since 2015 in the Seventh Regional Meeting of the Observatories for Human Resources in Health. Toronto Call to Action: Towards a decade of Human Resources in Health for the Americas stated that:

*“The Call to Action aims to mobilize institutional actors, both national and international, of the health sector and other relevant sectors and civil society, to collectively strengthen the human resources in health through both policies and interventions, in order to achieve the Millennium Development Goals and according to the national health priorities to provide access to quality health services for all the peoples of the Americas by the year 2015”* [5].

In the same vein, the Colombian Ministry of Health stated in 2018 that:

*“… Developing competencies in human talent from a broad health concept based on managing social determinants of health, a rights approach, interculturality, participation of patients and communities, protection of indigenous peoples, Afro-descendants, minorities and subjects of special protection, among others, is an imperative challenge to progressively guarantee the right to health provided for in the Statutory Law”* [6,7].

**Figure 1:**
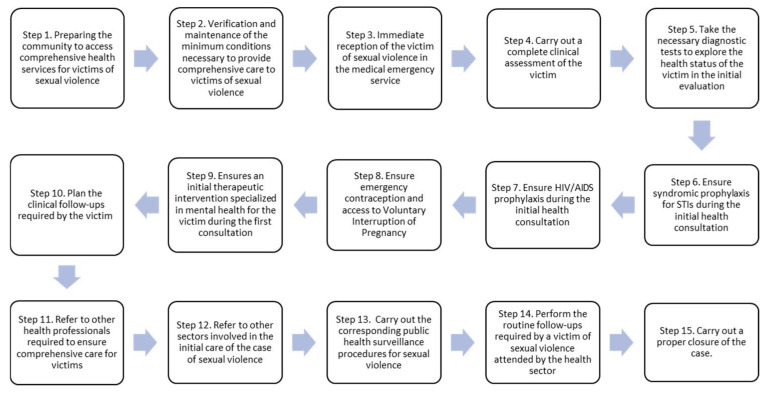
Comprehensive Health Care Protocol for Victims of Sexual Violence. Fundamental Steps in the Comprehensive Care of Victims of Sexual Violence.

Certainly, this challenge is key in the Colombian context, as the Care Protocol for survivors of sexual violence focuses on the role of medical and nursing staff, as can be seen in the following figure (Figure 1). 

Answers to these challenges are derived from complex and progressive processes that involve decisions made by agents and actors from all sectors. In particular, adjusting the supply of healthcare personnel for adequate response in terms of quantity, quality and relevance to immediate changes in healthcare demand can take several years. Such a time lag between the emergence of a need and its appropriate response creates temporary difficulties that are managed with available resources and capabilities. Therefore, while the supply of suitable human talent is adjusted in quantitative and qualitative terms, it is necessary to optimize the action of available human talent through management, care and service delivery models that are in line with the reality of the country and its territories, the configuration of multidisciplinary healthcare teams and appropriate use of available resources and technologies, among others [8] (p. 35).

Therefore, proper training of healthcare personnel is considered necessary and relevant here, particularly in regulatory, gender, sexual and reproductive matters, considering that they will likely be the first point of contact with women survivors of violence in relation to their right to health. For this reason, the role of healthcare personnel in the prevention and fight against gender-based violence is of great importance. 

Such needs of the healthcare personnel are certainly more important in neighborhoods such as Café Madrid and Colorados, located in the northern area of Bucaramanga, which is considered a marginal space in the city, leading to different situations of vulnerability among the population (mainly among the youngest). In this area, domestic violence rates among minors and young people are the highest at the national level.

Considering the above, this research aimed to determine knowledge and attitudes towards sexual and reproductive health and rights of adolescent girls among healthcare professionals working in the health centers of Café Madrid and Colorados.

## 2. Materials and Methods

This study was conducted using a qualitative descriptive methodology through in-depth interviews with healthcare professionals working in the health centers of Café Madrid and Colorados in Bucaramanga, Santander. 

The following question was asked: What is the meaning, structure and importance of an experience lived by a person (individually), group (jointly) or community (collectively) with respect to a phenomenon? The question is at the heart of the experience of the participant(s). The methodological approach is based on the following assumptions: (a) it aims to describe and understand the phenomena from each participant’s point of view and their collective perspective; (b) it is based on the analysis of specific discourses and topics, as well as the search for their possible meanings; (c) the researcher trusts their intuition and imagination to apprehend participants’ experience and contextualizes experiences in terms of temporality (time when they happened), space (place where they happened), corporality (physical persons who lived them) and relational context (ties generated by experiences) (Creswell, 1998; Alvarez-Gayou, 2003; and Mertens, 2005 (cited by Hernández, Fernández and Baptista, 2006)) [9].

The sample universe (*n* = 8) was composed of all healthcare personnel (physicians and nurses) working in the health centers of Café Madrid and Colorados in Bucaramanga, Santander. Inclusion criteria were being a healthcare professional of Café Madrid and Colorados health centers in Bucaramanga, Santander and working at the time of the study.

Participants were contacted by phone to explain the objective of the study and invite them to participate on a voluntary basis. Those who agreed to participate gave their verbal consent. Interviews lasted about 50 min each and were recorded and transcribed.

An interview script was used that included a battery of questions based on recommendations on adolescent sexual and reproductive health and rights made by the WHO [1] and DAPRE-Presidential Council for Women’s Equity—CedaVida Foundation [10]. In addition, sociodemographic variables such as environment, sex, age and professional experience were collected (Table 1).

A summative content analysis was carried out in four steps following the Braun, V. Clarke, Braun, Hayfield (2015) recommendations [11]. 

Firstly, two researchers read through the data set to become familiar with the material. After the first general reading of transcripts with the participation of two members of the research team, the identification of codes and categories was firstly made. This analysis of categories helped reach a consensus on the criteria of the coding process and thematic units of interest. Secondly, another team member compared identified categories. This triangulation process made it possible to test the level of consistency. After coding, the most significant units of analysis were extracted to identify the interrelationships among different themes. To review the themes, the most central characteristics and sub-elements of each theme were noted. Thirdly, the researchers went through the data set to see if and how each theme interacted with other themes, and if demographic variables played a role in each theme. Fourthly, the review of the themes was extended by highlighting the data set. 

The entire analytical process was carried out using QSR NVivo 11 software (University of Seville, Seville, Spain). 

A final report was prepared including the statements of each participant in the following way: “CM, number age” for the Café Madrid health center or “CO, number age” for Colorados health center. Descriptive analysis of the background information was carried out using numbers and percentages, as well as means and standard deviations.

### Ethical Considerations 

Participants were verbally informed about the study. The information included any potential risks of the study, voluntary participation and the right to refuse to answer any question or terminate the interview at any time. In addition, participants were informed that interviews would be recorded and quoted anonymously in publications and their identity would remain hidden at all times. In addition, participants provided their verbal consent. This study was approved by the Institutional Bioethics Committee of the Universidad de Santander and the ISABU Research Committee.

## 3. Results

### 3.1. Informants

The total sample consisted of 8 informants (4 women and 4 men), including 6 physicians and 2 nurses with a mean age of 39.1 (SD = 12) years. At the time of the interview, 37.5% (*n* = 3) reported being in a civil partnership as marital status. Additionally, 62.5% (*n* = 5) reported having children. The predominant socioeconomic stratum was 3. Regarding the relationship between informants’ parents, it was found that separated was the most common marital status. In addition, 62.5% (*n* = 5) reported being Catholic. One informant reported having experienced gender-based violence.

### 3.2. Technical and Legal Knowledge of Gender Mainstreaming/Gender Equity in the View of Health as a Fundamental Right

In this regard, our aim was to firstly explore the knowledge that healthcare professionals have about legal instruments related to sexual and reproductive rights and their relationship with the right to health in order to find out how this knowledge could influence care provided to girls and adolescents. Seven (7) informants claimed to have received training in this regard. When asked about specific knowledge, it was found that most healthcare personnel refer to the Comprehensive Healthcare Protocol for Victims of Sexual Violence issued by the Ministry of Health and Social Protection through training received on their own or received in their health centers as it is required for providing patient care. However, some of them also reported not having enough knowledge about it as they have not had to deal with this type of case.

*(CO2, 27 years old): "The truth is, well, more or less, because the truth is that I have never been touched by a case of sexual violence…"*.

*(CM3, 35 years old): “Yes, I am very aware of it because I am in outpatient clinics and the cases come that way”.*

*(CO1, 56 years old): "Yes, I worked 17 years in the emergency department (…) and there we were attending patients who were victims of sexual abuse, of course, we knew how to approach the patient from the emergency, we knew the discretion, we knew the protocols to have, to put into practice, to say medication, preventive issues, psychological, psychiatric, gynecological-obstetric management if it was the case".*

Regarding gender training, most of the health personnel reported having received it. Several of them indicated that their training in gender was related to that received on the care of survivors of sexual violence. The duration of this training varied widely, with some having received training lasting a couple of hours and others having received courses lasting several days and weeks.

(*CO1, 56 years old): “Yes, of course. We have to take a course on sexual abuse before joining the organization”.*

*(CM4, 23 years old): “A training I received at the university regarding violence and it lasted 1 h, another particular one that lasted about 8 h”.*

Regarding sexual violence, the health personnel stated that they have received training through courses on the care of survivors of sexual violence. They point out that this training sometimes focuses only on sexual violence and at other times joint training is given on other related topics. Most of the trainings have a duration of a couple of hours and others are up to 40 h.

*(CO3, 35 years old): "There was one of 40 h and the other one was a week long together with the one on gender violence".*

*(CO2, 27 years old): "Well, in the little course, in the course that I tell you about, the one on sexual violence—That one was sexual violence, physical violence and all types of violence".*

*(CM4, 23 years old): "Yes, the same day they explained about sexual violence and identification of vaginal injury, how to collect samples and other things...It was a congress that there was that dealt with all types of violence, including gender violence".*

Regarding intimate partner violence, the majority of the staff stated that they had not received training in this area in adults or adolescents. In some training sessions conducted in the work centers, reference is made to intimate partner violence when they study issues of intrafamily violence.

*(CM1, 36 years old): "I think that is what we do when a case of domestic violence or sexual violence is reported to the entities to which it should be reported and the notification to SIVIGILA".*

When asked about the knowledge of the importance and differences between sexual and reproductive rights, they identify them as part of the human rights that are violated mainly in girls, adolescents and women in general.

*(CO1, 56 years old): "Sexual rights are a person's right to express it freely, in their decision to be one gender or the other and the other is if they are already...or, better said, if they are homosexual, whether they are a woman or a man, they have the right to exercise their sexuality, that is the sexual right and the right to reproduction is the right to have the children they want".*

*(CM4, 23 years old): "…are the rights that a woman has to say about herself and her body".*

Some professionals also stated that these rights are not valued by women to assert their autonomy in the practice of these rights.

*(CO1, 56 years old): "…those are human rights, but many people do not know how to manage them, so if they are rights, absolutely, but there are people who do not know how to manage their rights, they do not know them, they do not value them and do not understand the meaning of why".*

### 3.3. Knowledge of Health Issues, Sexual and Reproductive Rights and Their Relationship with Gender Equity

It was found that the professionals recognize the importance of training on sexual and gender-based violence as a tool that facilitates the approach to the survivors and, despite having received training in this regard, the participants value the knowledge as an indispensable requirement for their labor articulation in the health sector.

*(CO1, 56 years old): "It was a prerequisite training course. It is a course that must be taken before, because it is required by the entity where you are going to work, now all entities require this preparation".*

*(CM3, 35 years old):"I took a course for the care of victims of sexual violence, which is required by most of the institutions”.*

Participating professionals recognize contraception as a sexual and reproductive right and, when asked about the choice of contraceptive method, it is clear that it is up to the women to decide the moment and choose the method they want, as long as it does not affect their health.

*(CM4, 23 years old): "I give them the freedom to choose which method they want to use. Then I evaluate the metabolic part before suggesting planning methods. I cannot recommend a contraceptive with a high hormonal load to patients with polycystic ovary disease, because I will end up harming them. I can suggest them, but since it is the patient's right, she can choose the method she wants".*

Regarding the form of care for survivors of sexual and gender-based violence, where their recognition of the issue could intervene in the processes of care for girls and adolescents, it was found that professionals recognize that survivors should have a differential and protected treatment, in a framework of respect with the objective of maintaining the dignity of the person.

*(CO1, 56 years old): "She is the first person to be seen when she arrives at an emergency department. If it were an outpatient department, she should be referred urgently to the emergency department”.*

*(CM2, 40 years old): "In the consultation, the patient should be received with the best possible treatment so that he/she feels more comfortable in the situation”.*

*(CM1, 36 years old):" When a user who refers to having suffered sexual violence enters, we pass her/him to the general practitioner by scheduling an immediate consultation, who makes a consultation and we follow a route”.*

Specifically on the issue of abortion, most of the professionals said they agreed only in cases where the grounds set out in the law are met; in general, they recognize that there is an institutional procedure (protocol) that must be followed and that it is established in the institutions where they work and that it complies with the framework of the law.

*(CO3, 35 years old): "when a girl arrives who does not want to have a baby, they are explained as such, the three grounds, they are given orientation anyway if they do not fall within the grounds they are sent to psychology and they end up doing as such the.... as they say, the training, no, the orientation, to know whether or not they are apt for abortion, the sentence is explained to them, the three grounds, what is the requirement in each ground, then they are oriented as such with psychology so that they can follow the process... one is obliged to give orientation, but that they agree, not in all cases”.*

Regarding the knowledge of the social conditions of the territory where the health centers are located, the participants recognize, especially the nursing staff, that the girls and adolescents attending consultations have the right to privacy, to exercise their sexual and reproductive rights and that this will facilitate the orientation on how to help them live a responsible sexuality.

*(CM4, 23 years old): "Most of the time dads do not allow girls to express their questions about e.g., contraceptives calmly and freely”.*

*(CO3, 35 years old): "the girls who come to us looking for guidance, they are mostly the ones who ask questions, they say that my friend's boyfriend does such and such, I don't know what...so we try to guide her directly so that things are clear, education is really very important”.*

Regarding the knowledge of the entities that provide support to survivors of sexual and gender-based violence, the professionals, especially nurses, are clear that there are entities that accompany and provide support and protection to survivors, either from the legal or social point of view, while there is more doubt among the other professionals.

*(CM1, 36 years old): "Yes. I have heard of some entities”.*

*(CM3, 35 years old): "My understanding is that to the police and emergency room”.*

*(CO3, 35 years old): "We have a directory that shows the patient what kind of support she has, the prosecutor's office, the family commissioner's office, the psychologist there, yes, the shelters, there are EPSs that provide them with shelters, so they are like alternatives for the client to avoid returning to the place where the aggressor is and not to return to be abused again and to show them alternatives, foundations that we have”.*

### 3.4. Barriers to Healthcare and Exercise of Sexual and Reproductive Rights in Girls and Adolescents 

When asked about the barriers observed by health personnel regarding the care and exercise of sexual and reproductive rights, all professionals indicated that they have not received training in personal skills for the approach to survivors of sexual violence and other gender-based violence, which makes it difficult to care for the survivors.

*(CM2, 40 years old): "This comes to us as poster-type information, but we do not receive information or training on how to address the issue in the consultation”.*

*(CO3, 35 years old): "-skills as such, no, the normal course”.*

In addition, the participants stated that it is difficult to identify violence in children; it is difficult because many of these acts are not identified by the children as some type of violence. Participants stated that it is further complicated because they often feel that they do not have the skills to address these cases.

*(CO2, 27 years old): "In itself in adult women it is difficult to address cases, in children it is worse, because as children let's say they do not have, how to tell you, that training in sexuality because they are children, then they sometimes, certain things that are obviously considered as sexual assault or abuse, they as such, as they have an immature mind, sometimes they do not interpret it that way and for them, as you know that children sometimes tend a lot to repeat what they say, they sometimes keep quiet because to them it seems normal”.*

*(CM3, 35 years old): "There are signs that are not so specific, but it can indicate that something is wrong. When children wet the bed, are afraid to go to the bathroom or are afraid of genital exploration. When genital exploration is performed, there is a lesion, a sexual behavior that, due to lack of knowledge or developmental stage, they do not have the capacity to refer to a sexual behavior. In addition, when a child says that he was touched here or that the uncle did such and such a thing to him, it is considered suspicious”.*

Another barrier identified is in relation to the free choice of the various contraceptive methods that exist; the administrative barrier limits the work of professionals because insurance companies only provide some family planning methods and this affects the final choice.

*(CO3, 35 years old): "…also to what the EPS offers, because not all EPS, at least these days, let's put it this way, …we have had this happen … they only have IUDs and quarterly injections, so the girl who arrives from that EPS cannot be offered anything else, because they have nothing else to give her, so it is up to you to manage that information, the effects and tell the patient, she wants these methods, or it is recorded in the history, that the EPS is only managing those methods and that the patient will start with another one for particular or things like that or that the patient is simply left without planning”.*

## 4. Discussion

The objective of this research was to determine the knowledge, attitudes and barriers of health professionals in health centers in the neighborhoods of Café Madrid and Colorados (Bucaramanga, Santander) with respect to the health, sexual and reproductive rights of girls and adolescents.

In relation to technical and legal knowledge regarding the meaning of the incorporation of the gender perspective/gender equity into the view of health as a fundamental right, this study found that the participants had received training mainly related to the Comprehensive Protocol for care of victims of sexual violence [12], either self-taught or through training received in the work centers with a varied intensity since it varied from a couple of hours to several days. This training is a positive aspect to highlight according to Arora et al. [13], who evaluated the knowledge, attitudes and practices of health providers before and after training on WHO guidelines and tools to improve the local context of healthcare for women survivors of violence, and the authors found that a training intervention combined with changes at the level of health systems gives better results in the knowledge, attitudes and practices of professionals who must care for women survivors of violence. This study mentions that to the extent that the objective is to build an effective and sustainable response of health professionals in the care of women survivors, it is not only necessary to limit this improvement in training but also to introduce changes and strengthening in the health system along with the implementation of the training intervention to create an adequate care environment, concluding that it is important not only to include content related to the identification of abuse and the response to it, but also to incorporate in these trainings of health professionals topics on attitudes, myths and misconceptions about violence against women, and that the trainings should be repeated over time to generate and maintain changes in the attitudes and practices of health professionals.

*This study identified that health professionals at some point in their professional practice received training on issues related to violence against girls, adolescents and women; however, it was found that some confusion persists, mainly related to the use of terminology to differentiate the type of violence that girls, adolescents and women may be suffering, hence the importance of integrating gender issues in the education of medical and health professions, as mentioned by Yang* [14], *as clarity on these issues in students and teachers promotes equal rights for people, and education on these issues should be considered as a professional discipline based on theory and practice as a tool for decision making*.

*Regarding knowledge on health issues, sexual and reproductive rights and their relationship with gender equity, this study found that professionals recognize the importance of the topic and that it provides them with elements for the approach to victims; this component of sensitization is related to what was mentioned by Ngoma* [15], *who noted that there is a statistically significant relationship between the level of knowledge of health professionals, receiving training on the topic of gender-based violence and reporting denunciation practices*.

Likewise, it was found among the responses that health professionals agree that women survivors of violence should have a differential and protected treatment that should be provided by the health professional with the objective of maintaining the dignity of the person. This is supported by Briozzo and Faúndes [16], who mention that medical and health professionals should be involved in the promotion of sexual and reproductive rights in women as a way of helping to provide the necessary resources to guarantee their right to the highest possible level of health.

Regarding the exercise of sexual and reproductive rights, this study shows that professionals agree that this should be lived freely, regardless of whether they are adolescents or older women, and that free exercise allows health professionals to provide information to women on responsible sexuality. Briozzo and Faúndes [16] also mention this when they state that empowering women about their sexual and reproductive rights is a key factor in the promotion and protection of their rights, regardless of whether they are adolescents, who should not be judged by their biological age, allowing them to make free, responsible and informed decisions about their health. 

Regarding the protection of women survivors of sexual and gender-based violence, in this study, health professionals recognize the importance of protecting women survivors and that, as part of this network of support and care, governmental and non-governmental institutions are fundamental in the protection of survivors’ rights, as highlighted by the Office of the United Nations High Commissioner for Human Rights (OHCHR) in its 2019 report on the protection of survivors of sexual violence [17], where it mentions that physical protection is the form of protection most commonly associated with criminal justice proceedings and is a responsibility that should be assumed, in a normal context, by national authorities. 

This study identified some barriers related to the development of personal skills to identify and attend to cases of sexual and gender-based violence, and it was identified that this situation may be due to the lack of familiarity of health professionals with the concepts and the topic of violence in general throughout their professional training, as evidenced by Briozzo and Faúndes [16], who mention that the most important role of health professionals, especially physicians, is to lead care teams by demonstrating their skills, attitudes and behaviors during daily interaction with patients. The authors highlight the challenge of building and developing such practice for the defense and promotion of sexual and reproductive rights, as a task for which health professionals do not receive training in the faculties.

With regard to the professional duties of health personnel and the right to abortion, the findings are in agreement with those of Diaz et al. [18] who point out that it is necessary for health personnel to receive training on the scope of the right to abortion, recalling that women have the right to a safe abortion and that although health professionals have the right to conscientious objection, this cannot be a tool to hinder a person from accessing a health service, as this would be an ethical and legal misconduct. In this regard, a previous study that related the influence of abortion law on the number of terminations performed with special attention to pregnancy terminations due to fetal defects comparing Poland and the United Kingdom showed that the radicalization of abortion law does not influence the decrease in the number of terminations due to fetal indications [19]. During the COVID-19 pandemic, in countries such as Italy, measures were incorporated to make the procedure more comfortable by not affecting health professionals in terms of indications and contraindications related to the procedure, information provided to the woman and ensuring the woman’s consent and understanding of the procedure [20].

The present study has limitations that should be taken into account when interpreting our findings. First, this study was carried out in two particularly interesting places in Bucaramanga (Colombia) and reflects the experiences of health professionals from Colombian health facilities. It is difficult to guarantee that the same results would be observed in other countries, since the backgrounds are different. Second, even though we used a universal sample, the sample is small and the generalizability of the results should be taken with caution.

## 5. Conclusions

Health personnel are trained to deal with cases related to sexual and reproductive rights and their relation to the right to health in the population under study. The training, on a good number of occasions, focuses mainly on the different care protocols established by the Ministry of Health and Social Protection of Colombia and adds a mixture of different topics that, among others, can range from gender to contraception. Moreover, it does not have a standard duration, so there are training courses that have lasted from a few hours to others that can last for days.

In relation to knowledge on health issues, sexual and reproductive rights and gender equity, health professionals know and are familiar with the related topics, due to their interest in receiving training in this regard. Likewise, this knowledge has allowed them to positively recognize specific topics such as the choice of contraceptive methods and abortion, care and differential and protected treatment of women when they are vulnerated and violated.

It is recognized that there is a lack of training in communication skills that could help in the care of patients who seek to inquire about their sexual and reproductive rights and especially patients who have had their rights violated. This lack is especially evident in the care of children whose communication is sometimes complex due to their evolutionary rhythms.

Finally, there are institutional barriers that permeate the professional work of health personnel in prescribing one or another contraceptive method for women, insofar as the woman’s choice of the specific method depends to a large extent on the offer available at any given time from the healthcare provider to which she belongs.

## 6. Recommendations

Given the results obtained in this study, it was recommended that the participating institutions design training sessions on the fight against gender violence that contain specific and defined topics that are addressed in depth. This is to avoid training that addresses a large number of topics in a short time that can generate confusion about the concepts and therefore limited attention.

It is also recommended that the participating institutions tend to provide health personnel with training that allows them to acquire personal skills, especially in childcare, such as welcoming, listening and containment based on the guidelines of the WHO and the Ministry of Health and Social Protection of Colombia. The above recommends to recognize and know how to offer an adequate response when it comes to girls and adolescents who may be at risk of gender-based violence.

Taking into account the interest in the subject of the participating health professionals and the social characteristics of the territory, it was recommended that the institutions hold refresher sessions that would allow the professionals to update their operational and legal knowledge on the subject.

Finally, it was recommended to the institutions participating in the study a greater dissemination of information related to the social and community support networks available to survivors of gender-based violence, not only among health professionals but also in the community so that they can make use of these networks.

## Figures and Tables

**Table 1:** Interview guide.

Part 1—Sociodemographic characteristics: Sociodemographic characteristics: age, sex, marital status, number of children, socioeconomic stratum, religion Self-recognition as a survivor of domestic violence or any type of violence (gender-based violence, intimate partner violence, inflicted by any person or friend)
Part 2—Semi-structured themes Technical and legal knowledge of gender mainstreaming/gender equity in the view of health as a fundamental right. Training in gender perspective (duration, mode of instruction) Training in sexual violence (duration, mode of instruction) Knowledge of the Protocol for Comprehensive Care for Victims of Sexual Violence Training in intimate partner violence, especially in adolescents Knowledge of health matters, sexual and reproductive rights and their relationship with gender equity Form of healthcare for survivor of sexual and gender-based violence Knowledge of social conditions of the health center where survivors attended Dissemination model used by entities supporting survivors of sexual and gender-based violence Barriers to healthcare and exercise of sexual and reproductive rights in girls and adolescents Selection of contraceptive methods by adolescents Positions regarding abortion and its requirements Knowledge of skills to care for survivors of sexual and gender-based violence in girls and adolescents

## Data Availability

Data are available from the authors (A.Y.R.-B and D.M.G.-C.).
